# *Saccharomyces cerevisiae*, bentonite, and kaolin as adsorbents for reducing the adverse impacts of mycotoxin contaminated feed on broiler histopathology and hemato-biochemical changes

**DOI:** 10.14202/vetworld.2021.23-32

**Published:** 2021-01-05

**Authors:** Toochukwu Ejiofor, Anthony Christian Mgbeahuruike, Chiamaka Ojiako, Ashang Micheal Ushie, Emmanuela Ifeoma Nwoko, Ibe Remigius Onoja, Toluwase Dada, Mulunda Mwanza, Magnus Karlsson

**Affiliations:** 1 Department of Agric Education, Faculty of Vocational Technical Education, University of Nigeria, 410002 Nsukka, Nigeria; 2 Department of Veterinary Pathology and Microbiology, Faculty of Veterinary Medicine, University of Nigeria, 410002, Nsukka, Nigeria; 3 Department of Human Kinetics and Physical Education, Faculty of Education, University of Nigeria, 410002 Nsukka, Nigeria; 4 Department of Animal Health, Faculty of Natural and Agricultural Sciences, North-West University, Mafikeng Campus, Private Bag X 2046 Mmabatho, 2735, South Africa; 5 Department of Forest Mycology and Plant Pathology, Swedish University of Agricultural Sciences, SLU, Box 7026, SE-75007 Uppsala, Sweden

**Keywords:** adsorbent, feed, mold, mycotoxin, pathology, *Saccharomyces cerevisiae*

## Abstract

**Background and Aim:**

*Saccharomyces cerevisiae*, bentonite and kaolin were used to reduce the adverse effects of mold-contaminated diet on broilers. The aim of the study was to evaluate the impact of *S. cerevisiae*, bentonite, and kaolin in reducing the adverse effects of mold (fungal) contaminated diet on broilers. Specifically, we investigated the histopathological, hematological, and serum biochemical changes associated with broilers fed mold-contaminated diets supplemented with these three adsorbents. We also isolated and identified the common fungal contaminants in the poultry feeds as well as the mycotoxins they produced.

**Materials and Methods:**

Hundred broilers (3-weeks-old) were randomly grouped into five dietary treatments, basal feed (negative control), feed contaminated with mold, mold-contaminated feed+*S. cerevisiae*, mold-contaminated feed+bentonite, and mold-contaminated feed+kaolin. The fungal contaminants in the feeds were isolated and molecularly identified while the mycotoxins in the feed where analyzed using high-performance liquid chromatography. Blood samples of birds from each group were analyzed for hematology and serum biochemistry. The liver, spleen, kidney, and bursa of Fabricius of the birds were excised and analyzed for histopathological changes.

**Results:**

The most common fungal contaminants in the feeds were *Penicillium* (33.3%) species, followed by *Aspergillus* species (22.2%). The mold-contaminated feed had the highest number of fungal contaminants, 55.6%, while the negative control (basal feed group) had none. Total aflatoxin and deoxynivalenol were high in the mold-contaminated feed (53.272 μg/kg and 634.5 μg kg, respectively), but these were reduced by the addition of adsorbents to the feed. The birds fed mold-contaminated feed had significantly (p<0.05) reduced red blood cell count counts, packed cell volume, and hemoglobin but increased white blood cell count compared to the negative control. Liver enzyme activity (alanine transaminase, aspartate aminotransferase, and alkaline phosphatase) and cholesterol concentration increased significantly (p<0.05) in the group fed mold-contaminated feed while the serum albumin and total protein decreased significantly (p*<*0.05) in comparison with the negative control. Adverse histopathological changes were observed in the liver, kidney, spleen, and bursa of Fabricius in the group fed mold-contaminated feed. Addition of *S. cerevisiae*, bentonite or kaolin in the mold-contaminated feed ameliorated these toxic effects.

**Conclusion:**

The observed histopathological lesions were consistent with mycotoxicosis in birds and were mild in the adsorbent treated groups. Kaolin had a higher protective effect against mycotoxicosis than the two other adsorbents.

## Introduction

The world poultry sector has undergone a huge expansion due to the demand for poultry meat, making it the fastest growing and most flexible of all livestock sectors [1]. The rapid expansion of this sector is partly as a result of quality feed formulation; and faster growth performance of birds caused by both genetic factors and better management conditions [2]. The Nigerian poultry industry has an annual growth rate of 2% and it has contributed so much to the development of the country in terms of food provision and employment generation. However, the industry faces a serious challenge due to poor processing of poultry feeds and feed contamination with mycotoxins [3]. In Nigeria, feed ingredients are bought locally by ­commercial feed companies during the production seasons and stored for a long period of time. The stored feed ingredients are used for feed production throughout the year. The long storage period predisposes the feed materials to fungal growth and mycotoxin contamination. Contamination of feed materials like grains by mycotoxins occurs either during the pre-harvest, harvest or during feed production, and storage. Mycotoxins are secondary metabolites of fungi found in grains and stored feed materials [4]. Some known mycotoxins that could have severe impacts on poultry health and performance are aflatoxins (AFs), ochratoxin A, T-2 toxins, zearalenone (ZEN), and deoxynivalenol (DON). High humidity and hot climatic ­conditions favor the growth of mycotoxin-producing fungi [5]. Some of the known mycotoxin producing fungi include *Aspergillus* which produces AF [6,7]. Fusarium species produce the mycotoxin ZEN which has estrogenic and immunotoxic properties [8,9]. Trichothecenes and DONs are produced by fungi of different genera such as *Trichothecium*, *Stachybotrys*, *Myrothecium*, *Cephalosporium*, *Trichoderma*, *Penicillium*, and *Fusarium* [8,9]. When mycotoxins are ingested in high doses, they can lead to acute clinical symptoms and severe damages to vital organs, damage to avian physiology as well as depression of the immune system of the affected birds [10]. Studies have shown that AFB_1_ could have a range of negative effects on poultry health, hematological, biochemical, and hepatorenal abnormalities [11-13].

The present study is significant because, there is need to search for locally available and cheap adsorbents, as means of decontaminating mycotoxin contaminated feed in low-income countries like Nigeria. Decontamination of poultry feeds can be done using chemical, physical, and biological approaches. Drying cereals before storage has been adopted because it reduces the high humidity content of feeds [14,15]. Furthermore, some bacteria that detoxify mycotoxins in the gut have been incorporated in feeds to prevent the absorption of such contaminants in the feed [15]. Other biological approaches involve the use of adsorbents or feed additives which bind mycotoxins, thereby preventing their absorption in the gastrointestinal tract [16]. Some commercially available adsorbents include aluminosilicates and esterified glucomannan derived from the cell wall of *Saccharomyces cerevisiae* [17]. However, locally available adsorbents such as fuller’s earth, bentonite, and activated charcoal have been reported to be effective in feed decontamination [18]. Some studies have shown the effects of different adsorbents on mycotoxin contaminated animal feed [19]. Although the effect of many adsorbents such as *S. cerevisiae*, bentonite, and kaolin on contaminated feed has been extensively studied, there is a paucity of information on their comparative effect on mold-contaminated diet in birds.

*S. cerevisiae* and other microorganisms have been reported to act as sequestering agents in the gastrointestinal tract of animals through the formation of mycotoxin-microorganism complex [20-22]. *S. cerevisiae* has been used in other studies as probiotic feed additives and for biodegradation of mycotoxin contaminated feeds [23,24]. Bentonite is structurally composed of several minerals, including montmorillonite, quartz, feldspar, volcanic glass, organic matter, and gypsum or pyrite. Bentonite has been used in AF decontamination of poultry feeds in other studies [19]. Kaolin is a plastic raw material consisting of the clay mineral kaolinite. Kaolin is formed through intensive weathering of rocks rich in feldspar (granite, arkose, certain types of orthogneisses, and migmatites). Due to its adsorbent capability and lack of primary toxicity, kaolin is used to prevent or ameliorate the adverse effects of many toxic agents. Kaolin clay minerals have been demonstrated to reduce diarrhea in animal production, improve feed conversion ratio, and health of many livestock species due to their specific adsorption potential of many feed mycotoxins [25].

The aim of the study was to evaluate the impact of *S. cerevisiae*, bentonite, and kaolin in reducing the adverse effects of mold (fungal) contaminated diet on broilers. Specifically, we investigated the histopathological, hematological, and serum biochemical changes associated with broilers fed mold-contaminated diets supplemented with these three adsorbents. We also isolated and identified the common fungal contaminants in the poultry feeds as well as the mycotoxins they produced.

## Materials and Methods

### Ethical approval

The broilers used in this experiment were handled in accordance with the revised version of the Animals Scientific Procedures Act of 1986 for the care and use of animals for research purposes. Furthermore, approval to conduct the study was obtained from the head of the Department of Microbiology and Pathology, Faculty of Veterinary Medicine, University of Nigeria Nsukka. The findings from this study are reported below with strict compliance to the procedures outlined in ARRIVE guidelines for reporting *in vivo* experiments in animal research [26].

### Study area and period

The experiment was conducted at the University of Nigeria Nsukka Agric farm. Nsukka is a small town in Enugu State located on latitude 6°51’21°N and longitude 7°51’ 24°N. The total land area is 1,810 km^2^ and it has a population of 309,633 with three senatorial zones [27]. The study was conducted from June to November 2019.

### Experimental birds

One hundred (3-weeks-old) Arbor acre broilers purchased from Okay Farms Nsukka were used for the study. The broilers were marked on the legs and each group was housed in a room-sized pen for acclimatization. During the period of acclimatization, the broilers were fed freshly prepared uncontaminated starter mash, formulated according to the revised version of the protocol for nutrient requirements of poultry [28]. Routine treatments and vaccinations were given to the broilers during the period of acclimatization. The broilers were moved to separate pens on the 4^th^ week and were allotted to five dietary treatments, with four replicates per treatment and five broilers per pen (replicate). They were exposed to 24 h light while the pens were maintained at environmental temperature. The birds were allowed free access to feed and water throughout the period of the experiment.

### Feed treatment and experimental design

The poultry feeds used in this study were purchased from a commercial feed dealer at Ogige Market Nsukka. All the feeds were from the same source and they all had the same date of production. The feeds were mixed together and placed in a large dark, water/air proof bag. Fungal growth and contamination of the feed were induced by sprinkling water on the contents of the bag until the feed was properly wetted and more than 30% moisture was obtained. The bag was sealed and stored in a room with a humidity of 50-60% and temperature range of 25-30°C, until mold growth was visibly observed. After 2 months of storage, the mold-contaminated feeds were divided into four different bags, the first bag was used as the positive control without further treatment. The other three bags were further treated with 2 g of bentonite/kg of the feed, 2 g of *S. cerevisiae*/kg of the feed, and 2 g of kaolin/kg of the feed, respectively. The locally prepared adsorbents were purchased at Joachim Laboratory Limited, University of Nigeria, Nsukka office. The feed additives were thoroughly mixed and the feeds were further stored for 2 weeks to allow the adsorbents to act properly on the mold-contaminated feeds. After 2 weeks, a freshly prepared feed, basal diet from the same source was purchased as a negative control and the feeds were served to the five groups of experimental birds for the entire period of the experiment.

### Mycotoxin measurement

Mycotoxin analysis was carried out using high-performance liquid chromatography (HPLC) (model 600 pump, 717 autosampler) with an in-line degasser and model 470 scanning fluorescence detector. Mycotoxin was extracted as described in Bhatti *et al*. [19]. Briefly, approximately 10 g of each feed sample was ground with mortar and pestle and transferred to extraction tubes containing water and acetonitrile (20:80%, v/v). The solution was placed in a rotary shaker for 45 min for proper mixing and later subjected to the extraction process. For each sample, 1 g of sodium chloride and 20 mL of n-hexane were added to the tube and thoroughly mixed together. The mixture was filtered using filter paper (Schleicher and Shuell, 597½) and the filtrate was centrifuged for 10 min at 4000 g. The lower methanol phase was used for immune affinity cleanup while the upper hexane phase was discarded. About 40 mL of de-ionized water was diluted with an aliquot (1 mL) of the extract and mixed thoroughly. The resulting solution was purified on immunoaffinity columns (VicamAflaTest, VICAM, 313 Pleasant Street, Watertown MA, USA.) and the extract was further analyzed using reverse-phase HPLC (Shimadzu Corp.) with isocratic elution and fluorescence detection, after post-column derivatization with bromine by KOBRA CELL® (Rhone Diagnostics, Glasgow UK). The total AF (AFB_1_+AFB_2_+AFG_1_+AFG_2_) in the samples was measured alongside the mycotoxin, DON).

### Isolation of the fungal contaminants

The fungal contaminants in the feed were isolated by the dilution plate technique [29]. One gram of feed sample from each treatment group was suspended in 9 mL of sterile distilled water and mixed thoroughly for 2 min by hand inversion. Approximately 0.1 mL aliquot of the suspension was plated in triplicates on potato dextrose agar (PDA) and the plates were incubated at 28°C. After 48 h of incubation, the colonies in each plate were counted and recorded as the fungal load per sample and the colony-forming unit/g was calculated for each sample. Each fungal colony from the mixed culture in each plate was carefully picked with a sterile toothpick and transferred again into sterile and freshly prepared PDA plates for final purification [30]. The fungal colonies were isolated based on their morphological characteristics, such as color of isolate and physical appearance on the media [31].

### Molecular identification of the fungal contaminants

#### DNA extraction

Genomic DNA extraction from the fungal isolates was done using the Zymo Research kit (Zymo-Research fungal/Bacterial Soil Microbe DNA, D6005, USA), following the procedure described in other studies [32]. Briefly, a loopful of the fungal spores from 5 to 7 days old culture was scooped into the Bashing Bead^™^ lysis tubes and 750 μL of the lysis solution was added to the tubes. The tubes were beaten in a bead beater at maximum speed for 14 min and centrifuged in a micro-centrifuge at 10,000× *g* for 1 min. Four hundred microliters of the supernatant were transferred into a collection filter tube which was centrifuged at 10,000× *g* for 1 min after which 1200 μL of the binding buffer was added to enhance the binding of the toxins to the filter column. The mixture was further centrifuged in the column in the collection tube and the flow-through discarded. About 200 μL and 500 μL of pre-washed and washed buffers were added separately to each column, respectively, and centrifuged at the same conditions. Finally, 100 μL of the DNA elution buffer was added to the column after washing and centrifuged to elute the DNA.

### Polymerase chain reaction (PCR) amplification of genomic DNA and sequencing

The amplification of the Internal Transcribed Spacer Region (ITS rDNA) of the fungal isolates from the feed was carried out with PCR using the ­universal primers (Table-1) supplied by Inqaba biotechnical Industrial (Pty) Ltd. (Pretoria, South Africa). The amplification parameters included an initial denaturation at 94°C for 5 min, followed by 30 cycles of 94°C for 35 s, annealing at 58°C for 30 s, and extension at 72°C for 25 s, with a final extension at 72°C for 10 min. The PCR amplicons were analyzed by electrophoresis on 1% (w/v) agarose gel and visualized under UV light [33]. Sequencing of the purified PCR products was done at Inqaba Biotechnical Industrial (Pty) Ltd, Pretoria, South Africa with PRISM™ Ready Reaction Dye Terminator Cycle Sequencing Kit, using the dideoxy chain termination method. The sequenced samples were electrophoresed with a model ABI PRISM^®^ 3500XL DNA Sequencer (Applied BioSystems, Foster City, CA, USA) following the manufacturer’s instructions. Finch TV software version 1.4.0 (Geospiza, Inc. Seattle, WA, USA) was used for the analysis of chromatograms, resulting from the sequencing. The consensus ITS rDNA sequences obtained were Blasted in the National Center for Biotechnology (NCBI) database (www.ncbi.nlm.nih.gov) with the Basic Local Alignment Search Tool for homology to identify the probable organisms in question [34]. The sequences were later deposited in the GenBank for accession number allocation.

**Table-1:** Primer sequences.

ITS 1-Forward primer	TCC GTA GGT GAA CCT GCG G
ITS 4-Reverse primer	TCC TCC GCT TAT TGA TAT GC

Primer sequences used for the amplification of the internal transcribed region of the fungal gene

### Sample collection from the experimental birds

The sampling from the experimental birds was done during the 10^th^ week of the experiment. Three birds per pen were randomly selected; blood samples were collected from the jugular veins of the selected birds into heparinized tubes and non-heparinized tubes for hematology and serum biochemistry, respectively. They were humanely sacrificed and dissected for collection of liver, kidney, spleen, and bursa of Fabricius samples. Two samples each were collected for each organ and were fixed in 10% buffered formalin for histopathology.

### Hematological and serum biochemical analysis

The packed cell volume (PCV) was determined using the Microhematocrit method while the red blood cell (RBC) count and total white blood cell (WBC) count were determined using the hemocytometer method [35]. Hemoglobin (Hb) concentration was determined using Drabkin’s reagent assay method for Hb concentration. The serum biochemical parameters were determined using Quimica Clinica Aplicada test kits (Quimica Clinica Aplicada, Spain) and a spectrum Lab 21A spectrophotometer (Spectrum Lab, England). The activity of the liver enzymes, alkaline phosphatase (ALP), alanine transaminase (ALT), and aspartate aminotransferase (AST) was determined using commercial kits (ALP [AP307], AST [AS101], and ALT [AL100] - Randox, United Kingdom) and a spectrophotometer. ALP was measured at 405 nm while AST and ALT were measured at 546 nm. The total serum protein and serum globulin were obtained, following the procedure detailed in Rezende *et al*. [36]. The serum cholesterol was determined by enzymatic colorimetric method [37].

### Histopathology

Histopathological examination of the excised organs was carried out as described by Bancroft *et al*. [38]. Briefly, the liver, kidney, spleen, and bursa of Fabricius were obtained from the dissected birds at the end of the experiment (10^th^ week) and fixed in 10% neutral buffered formalin. The fixed tissues were then passed through several concentrations of alcohol and xylene before embedding in paraffin wax. The embedded tissues were sectioned using a rotary microtome at a thickness of 5 mm. The sections were picked and floated on a water bath and then picked with a pre-labeled slide. The slides were dried on a hot plate at a temperature of 5-10^°^C above the melting point of wax, dewaxed with xylene, and hydrated through descending grades of alcohol before staining with hematoxylin and eosin method to demonstrate general tissue structure. The slides were then mounted on coverslips with DPX devoid of air bubbles. The specimens were examined and photomicrographs were captured using a Motic Images plus 2.0 digital cameras (Motic China Group Ltd. 1999-2004).

### Statistical analysis

Statistical analysis of the data was performed using SPSS statistics 23.0 version software (IBM Corp, USA). Multiple comparisons were performed using one-way analysis of variance, followed by *post hoc* test and significant differences were determined at p<0.05.

## Results

### Biodiversity of the fungal contaminants in the feed

Initial culture of the feed samples produced fungi with mixed morphologies. Pure culture of each fungal isolate led to the isolation of a plethora of fungi with discernible identity. Six major genera of fungi were found in all the feeds; *Epicoccum*, *Rhizomucor*, *Pichia*, *Penicillium*, *Aspergillus*, and *Beauveria* (Table-2). The mold-contaminated feed, contaminated feed supplemented with *S. cerevisiae*, and contaminated feed supplemented with kaolin had the highest representation of fungal contaminants (55.6%) each (Table-2). The culture samples from the basal feed produced no fungal contaminant. Sequencing of the fungal ITS region for identification to the species level and blast search at the NCBI revealed three species of *Penicillium*; *Penicillium bilaiae*, *Penicillium crustosum*, and *Penicillium glabrum*; two species of *Aspergillus*; *Aspergillus niger* and *Aspergillus Fumigatus;* and one species each for the other ­fungal isolates (Table-3). The sequences of the identified fungi were deposited at GenBank in NCBI with accession numbers as indicated in Table-3.

**Table-2:** Biodiversity of the fungal contaminants in the feed.

Fungal contaminants	Feed treatments				

	Fresh feed	Con feed	Con feed +B	Con feed +*S*	Con feed +K
*Epicoccum sorghi*	x	✓	✓	✓	✓
*Rhizomucor pusillus*	x	✓	✓	✓	✓
*Pichia farinosa*	x	✓	x	x	✓
*Penicillium bilaiae*	x	✓	x	x	✓
*Penicillium crustosum*	x	x	✓	✓	x
*Penicillium glabrum*	x	x	x	x	✓
*Aspergillus fumigatus*	x	x	x	✓	x
*Aspergillus niger*	x	✓	x	x	x
*Beauveria felina*	x	x	x	✓	x

✓ =Fungus present, x=Fungus not present. Fungi were isolated based on their morphological characteristics and molecularly identified by sequencing of the ITS region. Con feed=Contaminated feed, Con feed+S=Contaminated feed+*Saccharomyces cerevisiae*, Con feed+B=Contaminated feed+bentonite, Con feed+K=Contaminated feed+kaolin

**Table-3:** Fungi identified by ITS sequencing and their GenBank accession numbers.

Sample identity	Fungi identity	Accession No	% similarity
Seq01a	*Epicoccum sorghi*	KC106688	79
Seq02a	*Rhizomucor pusillus*	KY583064	99
Seq03a	*Pichia farinosa* var*. farinosa*	FJ196777	99
Seq04a	*Penicillium bilaiae*	KU574710	99
Seq04c	*Penicillium crustosum*	KT192315	98
Seq04d	*Penicillium glabrum*	EU128622	99
Seq12	*Aspergillus fumigatus*	MH345857	99
Seq09	*Aspergillus niger*	KR085975	99
Seq14	*Beauveria felina*	KF993397	89

Fungi identified by PCR amplification of the Internal Transcribed Spacer Region (ITS rDNA) of the fungal isolates from the feed using the universal primers ITS1 and ITS4 followed by sequencing and blast search at NCBI database. Fungal sequences were deposited at GenBank with the accession numbers on the table

### Mycotoxin concentration in the feed

The basal diet had the lowest concentration of total AF (16.874 μg/kg) while the mold-contaminated feed without any adsorbent had the highest concentration (53.272 μg/kg). The total AF concentration was considerably reduced in the feeds supplemented with adsorbents. The contaminated feed treated with kaolin had the highest reduction in AF, followed by the bentonite treated feed (Table-4). The concentration of DON was lowest in the basal feed (26.48 μg/kg) and highest in the mold-contaminated feed (634.5 μg/kg). The mold-contaminated feed treated with kaolin had the highest reduction of DON compared to the other adsorbents treatments (Table-4).

**Table-4:** Mycotoxin concentration in the different feed treatments.

Treatments	Total aflatoxin (µg/kg)	DON (µg/kg)
Basal diet	16.874	26.48
Mold-contaminated feed	53.272	634.5
Mold-contaminated feed+SC	33.04	409.5
Mold-contaminated feed+B	23.674	332.925
Mold-contaminated feed+K	18. 154	251

DON=Doxylevanelone, SC=*Saccharomyces cerevisiae*, B=Bentonite, K=Kaolin

### Histopathology

**Figure-1:**
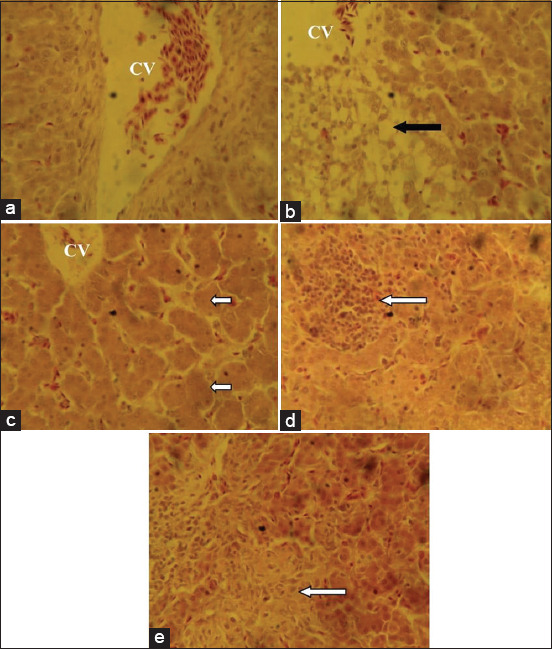
Photomicrograph of liver sections from the experimental groups: (a) Birds fed basal feed, showing normal histological features; (b) birds fed mold-contaminated feed showing centric-lobular degeneration and necrosis/fatty changes in hepatocytes (black arrows); (c) birds fed mold-contaminated feed+Saccharomyces cerevisiae, showing distorted and acinar arrangement of hepatocytes (short arrows) while (d) birds fed mold-contaminated feed+bentonite; and (e) birds fed mold-contaminated feed+kaolin, showing nodular areas of lymphoid cell accumulation (white arrows). See the CV (H and E, ×400). CV=Central vein.

**Figure-2:**
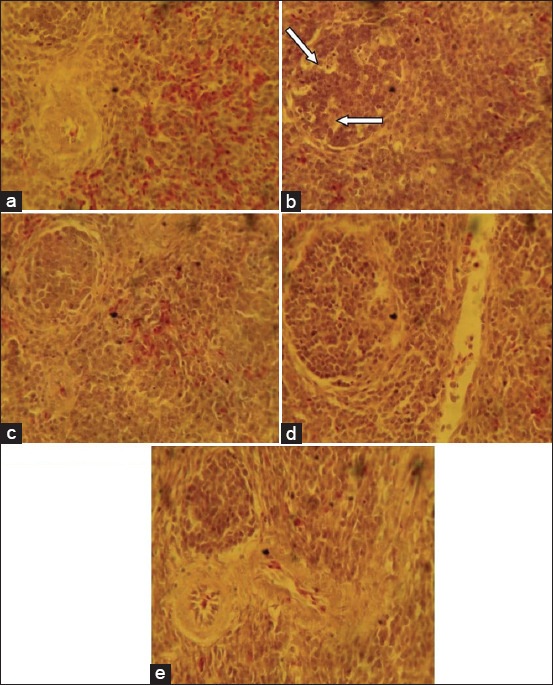
Photomicrograph of kidney sections from the experimental groups: (a) Birds fed basal feed, showing normal histological features; (b) birds fed mold-contaminated feed, showing mild degeneration of the tubular epithelium (arrows), while (c) birds fed mold-contaminated feed+Saccharomyces cerevisiae; (d) birds fed mold-contaminated feed+bentonite, and (e) birds fed mold-contaminated feed+kaolin show no observable histological change. See the GM (H and E, ×400). GM=Glomerulus.

**Figure-3:**
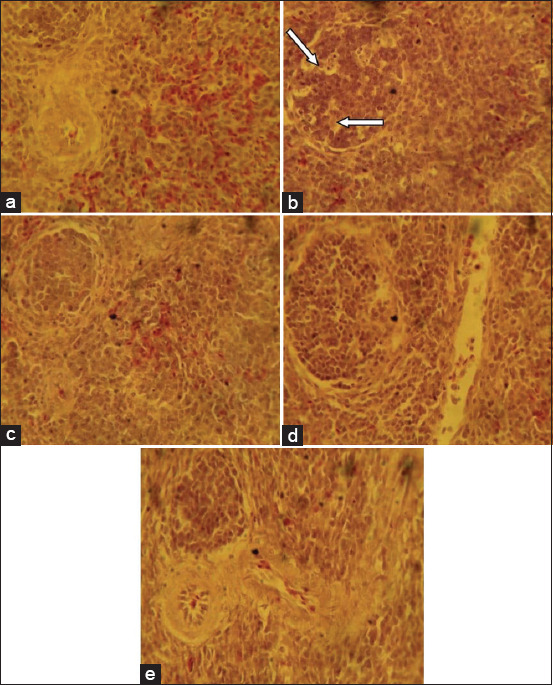
Photomicrograph of spleen sections from the experimental groups: (a) Birds fed basal feed, showing normal histological features, (b) birds fed mold-contaminated feed, showing cellular depletion and degenerative changes in the white pulp of the germinal center (arrows), (c) birds fed mold-contaminated feed+Saccharomyces cerevisiae, (d) birds fed mold-contaminated feed+bentonite, and (e) birds fed Mold-contaminated feed+kaolin show no observable histological change (H and E, ×400).

**Figure-4:**
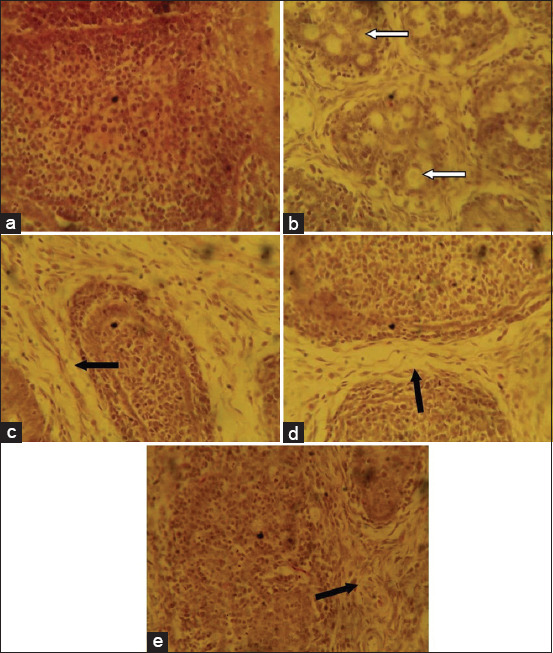
Photomicrograph of the bursa of Fabricius sections from the experimental groups: ( a) Birds fed basal feed, showing normal histological features; (b) birds fed mold-contaminated feed, showing mild-to-moderate lymphocytic depletion in their follicles (arrows), (c) birds fed mold-contaminated feed+Saccharomyces cerevisiea, (d) birds fed mold-contaminated feed+bentonite, and (e) birds fed Mold-contaminated feed+kaolin, show edema and widened interfollicular septa (black arrows) (H and E, ×400).

The liver sections from the birds fed with the basal feed showed normal histological features while birds that ate the mold-contaminated feed showed centric-lobular degeneration, necrosis, and fatty changes in the hepatocytes (Figure-1).The group fed mold-contaminated feed supplemented with *S. cerevisiae* showed distorted and acinar arrangement of hepatocytes while the group fed mold-contaminated feed supplemented with bentonite or kaolin showed nodular areas of lymphoid cell accumulation (Figure-1). The kidney sections from the group fed mold-contaminated feed showed mild degeneration of the tubular epithelium while all the other treatment groups had normal histological features of the kidney (Figure-2). The sections of the spleen from the group fed the basal feed showed normal histological features compared to the group fed mold-contaminated feed which showed cellular depletion and degenerative changes in the white pulp of the germinal center while the groups fed mold-contaminated feed supplemented with either *S. cerevisiae*, bentonite or kaolin showed no observable histological changes (Figure-3). The bursa of Fabricius of the group fed basal feed showed normal histological features compared with the group fed mold-contaminated feed that showed mild to moderate lymphocytic depletion in their follicles. However, all the adsorbent treated groups showed edema and widened interfollicular septa of the bursa of Fabricius (Figure-4).

### Hematology and serum biochemistry

**Figure-5:**
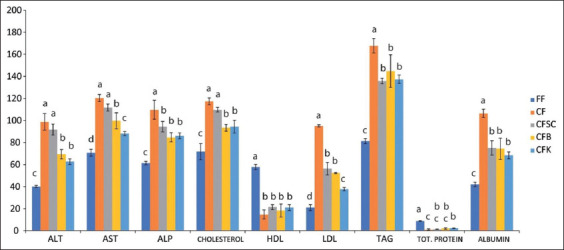
Serum biochemistry. Serum biochemical analysis of birds treated with mold-contaminated feed with or without adsorbent supplementation. (a) FF=Fresh feed/basal feed (negative control), (b) CF=Mold-contaminated feed, (c) CFSC=Mold-contaminated feed supplemented with Saccharomyces cerevisiae, (d) CFB=Mold-contaminated feed supplemented with bentonite (e) CFK=Mold-contaminated feed supplemented with kaolin. ALT=Alanine aminotransferase; AST=Aspartate aminotransferase; ALP=Alkaline phosphatase; HDL=High density; LDL=Low density; TAG=Total Protein.

Table-5 shows the results of the hematological analysis of the experimental birds. The group fed mold-contaminated feed had a significant (p<0.05) decrease in RBC count, PCV, hemoglobin concentration, and increase in WBC count compared to the control group that ate the basal feed. Furthermore, the serum liver enzyme activity (ALT, AST, and ALP) and cholesterol concentration increased significantly (p<0.05) in the group fed mold-contaminated feed while the serum total protein and albumin ­concentration decreased significantly (p<0.05) in the same group (Figure-5). Inclusion of adsorbents in the mold-contaminated feed led to an increase in PCV, hemoglobin concentration, RBC count, and decrease in the WBC count. A decrease in ALT, AST, and ALP activity and cholesterol concentration and an increase in serum total protein and albumin concentration were observed in the adsorbent treated groups compared with the group fed mold-contaminated feed. However, the addition of kaolin and bentonite to the feed restored the hematological and serum biochemical parameters of the broilers (Table-5 and Figure-5).

**Table-5:** Hematological parameters of broilers fed mold-contaminated feeds with or without *Saccharomyces cerevisiae*, bentonite or kaolin.

Treatments	PCV (%)	Hb (g/dl)	RBC×106/L	WBC×103/L
Basal diet	35.00±0.58^a^	11.60±0.20^a^	11.13±0.38^a^	9.83±0.26^a^
Mold treated feed	22.00±1.15^c^	6.60±0.20^c^	6.09±0.21^c^	17.50±0.61^b^
Mold treated feed +SC	24.33±1.20^b,c^	7.03±0.15^b,c^	7.21±0.21^b,c^	16.80±0.26^b^
Mold treated feed +B	26.00±0.58^b^	7.10±0.10^b,c^	8.08±0.21^b^	14.60±0.40^c^
Mold treated feed +K	26.67±0.8^b^	7.97±0.49^b^	8.34±0.15^b^	13.60±0.26^c^

Means (mean±SEM) on the same row with different superscript are significantly different (p<0.05). PCV=Packed cell volume, Hb=Hemoglobin, RBC=Red blood cell, WBC=White blood cell, SC=*Saccharomyces cerevisiae*, B=Bentonite, K=Kaolin

## Discussion

The commercial poultry feed used in the current study was treated in a way that promoted visible growth of fungal molds, and partly mimic the storage conditions in commercial broiler production in the tropics. The humidity introduced by sprinkling of water on the feed and the prevailing hot climate helped the growth of mycotoxin producing fungi [5]. The fungal growth induced the production of the identified mycotoxins; AF and DON. A very high concentration of AF was recorded in the mold-contaminated feed without adsorbent supplementation. A similar trend was observed for DON mycotoxin in the mold-contaminated feed. However, the addition of the three adsorbents to the feeds caused a drastic reduction of both mycotoxins. Kaolin had the highest mycotoxin reducing ability, followed by bentonite. Although not much is known in the literature on the mycotoxin degrading ability of kaolin, kaolin and bentonite are commonly used adsorbents because they lack primary toxicity. Their structural composition gives them a large surface area and better absorption capacity. *S. cerevisiae* showed the least efficacy in the decontamination of the mold-contaminated feeds. *S. cerevisiae* acts as a sequestering agent and forms mycotoxin-microorganism complex, and this excellent attribute makes it a good agent for biodegradation of mycotoxin in contaminated feeds [20,24]. A comparative study of the mycotoxin decontaminating ability of bentonite, activated charcoal and fuller’s earth showed that bentonite had the highest efficacy and was able to lower the AF concentration of the feed from 120 ± 38 μg/kg to 15 ± 5.0 μg/kg [18]. *S. cerevisiae* was reported to bind >60% of AFs in a study where the binding efficacy of different yeast isolates was determined. However, the microbes were reported to exhibit lower reducing activity compared to bentonite [39]. The basal feed had total AF level below the 20 μg/kg recommended by the United States food and Drug Administration but the concentration of DON in the feed was a little above this recommended level.

A wide range of fungal species from six different genera were found in the mold treated feed samples and also in the mold treated feeds supplemented with adsorbents. Two known mycotoxin producing fungi, *Penicillium* and *Aspergillus* species were prominent in the feed samples. *Penicillium* species are known to produce the mycotoxin; trichothecenes, including DON [8,9]. *Aspergillus* species are good producers of AFs [16]. In a previous study, feed samples exposed to the same conditions were contaminated with up to 120 μg/kg AFs (mainly AFG_1_ and AFB_1_), produced by a range of different *Aspergillus* [18]. It is, therefore, not surprising to observe high concentration of AF and DON in the feed samples. These mycotoxins may be the primary cause of the observed pathological changes in the birds.

Hemostasis and damage to blood system are major adverse effects associated with mycotoxicosis in animals [40]. In the present study, the mold-treated diet increased the WBC level of the birds by 44% but the RBC, Hb, and PCV were lowered by 55, 56, and 62%, respectively. The reduction in RBC, PCV, and Hb levels may be an indication of anemic condition due to alteration of the hemopoietic processes by the mycotoxin [41]. The anemia may have occurred due to increased erythrocyte destruction in the hematopoietic organs, or the dysfunction of enzyme activities involved in heme biosynthesis [41]. The kaolin feed additive slightly improved the PCV, RBC, and Hb levels and decreased the WBC counts. The addition of bentonite also showed a positive effect on PCV, RBC, and WBC levels. This result is supported by data from supplementing AF-contaminated feed with 0.2% hydrated sodium calcium alumino silicate and 0.2% multi-mycosorbents [42]. However, in the current study, using *S. cerevisiae* as a feed additive had the least positive effect with regard to the blood parameters analyzed. Previously, certain *S. cerevisiae* strains were shown to bind AFB_1_ but there was a significant variation between strains that may explain the result in the current study [20]. The activity of AST increased by 42% while the ALT and ALP increased by 43 and 44%, respectively, in the group fed mold-contaminated feed. The observed increase in the activity of the liver enzymes, AST, ALT, and ALP may be an indication of liver mycotoxicosis. Increases in activity of the liver enzymes have been reported previously in broilers fed AF-contaminated feed [18]. AFB_1_ contaminated diets were reported to decrease levels of total cholesterol in ducklings [41]. However, in the present study, the cholesterol level was increased by the mold-contaminated feed. The variation in the results could be a result of species differences. While Filho *et al*. [41] used ducklings in their study, the present study was conducted using broilers.

The histopathological changes of the birds fed mold-contaminated feeds ranged from centric-lobular degeneration, necrosis, and fatty changes of the hepatocytes to mild degeneration of the tubular epithelium of the kidney. The spleen and bursa of Fabricius of the group fed mold-contaminated feeds showed moderate changes in the white pulp and follicles, respectively. The observed histopathological changes in these organs were consistent with the previous reports on the effects of mycotoxins in birds [18,43]. Birds experimentally treated with mycotoxin-contaminated feeds and those treated with contaminated feeds protected by adding 1% Mycofix Plus showed comparable results. Histopathological sections of organs from both groups of birds showed extensive lesions in the positive control group while the Mycofix practically reduced the lesions in the protected group [26]. The liver histopathological changes were corroborated with the result from the serum biochemistry. Mycotoxins such as AFs have been reported to cause severe adverse effects in the liver, kidney, and lymphoid organs such as the spleen and bursa of Fabricius [10,44]. However, the various degrees of histopathological changes in the organs of birds fed the adsorbent treated feed showed the suppressive effects of the feed additives against the mycotoxins. This is consistent with similar reports on the ameliorative effects of *S. cerevisiae* cell wall and bentonite on broilers fed AF-contaminated feed [16]. Bentonite has been reported to have hepatoprotective and nephroprotective effects against mycotoxicosis [18]. Furthermore, reports have shown that bentonite, fuller’s earth, and activated charcoal were able to prevent liver pathologies in birds fed AF-contaminated feed [18].

## Conclusion

The results from this study show that kaolin has a more protective effect against mold-contaminated feed induced mycotoxicosis in broilers, followed by bentonite and lastly, by *S. cerevisiae*. The findings from this work could be useful to feed producers and poultry farmers as the adsorbents can be used as feed additives to enhance the efficiency and performance of birds if mycotoxin is suspected in feeds.

## Authors’ Contributions

TE contributed in conceptualizing the study, designing the work and supervision. ACM conceptualized the study, supervised it, helped in data analysis and wrote the manuscript. CO and AMU did part of the lab work. EIN helped with data analysis. IRO did the histopathological analysis, TD and MM did the molecular studies. MK did the data analysis and helped in manuscript preparation. All authors read and approved the final manuscript.
